# From local control to systemic management: radiotherapy and sequential methotrexate therapy in an adult BRAF-negative hypothalamic-pituitary Langerhans cell histiocytosis – a case report with 15-month follow-up

**DOI:** 10.1186/s12902-026-02509-2

**Published:** 2026-08-25

**Authors:** Chi Feng, Zhiming Chen, Liang Chen, Jun Qin, Desheng Wei, Lei Mou

**Affiliations:** 1 Department of Neurosugery, Taihe Hospital, Hubei University of Medicine, Hubei, China; 2 Hubei Provincial Clinical Research Center of Central Nervous System Repair and Functional Reconstruction, Hubei, China

**Keywords:** Langerhans cell histiocytosis, Hypothalamic-pituitary region, Central diabetes insipidus, Hyperprolactinemia, Radiotherapy, Methotrexate

## Abstract

**Background:**

Langerhans cell histiocytosis (LCH) is a rare clonal neoplastic disorder characterized by the proliferation of CD1a+/Langerin+ dendritic cells. Hypothalamic-pituitary region (HPR) involvement in adults—particularly when followed by multisystem progression after local therapy—remains poorly characterized. We report a 35-year-old female with HPR-LCH who achieved durable local metabolic remission after radiotherapy but later developed systemic progression, which was successfully managed with systemic chemotherapy.

**Case presentation:**

A 35-year-old woman presented with progressive memory decline, polydipsia, polyuria, 15-kg weight gain over six months, and one year of amenorrhea. Endocrine evaluation revealed central diabetes insipidus (CDI) and hyperprolactinemia (prolactin 94.19 ng/mL). Pituitary MRI demonstrated a 2.0 × 1.4 cm enhancing hypothalamic mass with pituitary stalk thickening. A pterional craniotomy was performed for histopathological sampling, and immunohistochemistry confirmed LCH (CD1a+/Langerin+/S100+); BRAF V600E mutation was negative by quantitative PCR. The patient received involved-field intensity-modulated radiotherapy (IMRT, 22 Gy/11 fractions) targeting the HPR lesion. At 15-month follow-up, 18 F-fluorodeoxyglucose positron emission tomography-computed tomography (18 F-FDG PET-CT) demonstrated complete metabolic remission of the hypothalamic lesion, with resolution of memory and mood symptoms. However, new progressive skull bone destruction, skin ulcers, and pulmonary nodules emerged. She was subsequently treated with a full initial course of high-dose methotrexate (MTX 9 g IV weekly for 3 weeks, 12 g weekly for 2 weeks, and 16 g weekly for 2 weeks) at a Peking Union Medical College Hospital-affiliated institution, achieving significant improvement in skin ulcers, bone pain, and galactorrhea. A second consolidation course is scheduled.

**Conclusion:**

This case illustrates that involved-field IMRT can achieve durable local control of HPR-LCH even in BRAF V600E-negative cases. However, local control does not equate to systemic cure; close surveillance for extracranial progression is essential, and timely systemic chemotherapy (e.g., high-dose MTX) is critical for managing multisystem disease. Lifelong endocrine surveillance remains mandatory.

**Supplementary Information:**

The online version contains supplementary material available at https://doi.org/10.1186/s12902-026-02509-2.

## Background

Langerhans cell histiocytosis (LCH) is a rare clonal neoplastic disorder characterized by the proliferation of CD1a+/Langerin+ dendritic cells. While primarily affecting children (incidence: 4–5 per million), adult-onset LCH is exceptionally uncommon (0.5–1 per million), with isolated hypothalamic-pituitary region (HPR) involvement representing < 5% of adult cases [1, 2].

The hypothalamic-pituitary axis is a critical yet vulnerable site for LCH infiltration. Central diabetes insipidus (CDI) is the hallmark presentation (75–90% of cases), often preceding other endocrine deficiencies (e.g., anterior hypopituitarism, hyperprolactinemia) by months to years [3]. MRI typically reveals pituitary stalk thickening (> 3 mm) or contrast-enhancing masses in the infundibulum or mammillary bodies, mimicking lymphocytic hypophysitis, germinomas, IgG4-related disease, or neurosarcoidosis [4].

The diagnostic challenges of HPR-LCH stem from the deep location, proximity to vital structures, and restrictions regarding biopsy specimen size [5]. Pathological confirmation remains imperative, requiring immunohistochemical demonstration of CD207 (Langerin) and CD1a positivity [6].

Therapeutic standardization remains elusive in adult HPR-LCH, with current guidelines extrapolated from pediatric data or multisystem LCH studies. For localized HPR disease, involved-field radiotherapy (15–24 Gy) has shown > 80% local control rates [7, 8]. For multisystem disease, systemic therapies including cladribine, high-dose cytarabine, methotrexate-based regimens, or BRAF inhibitors (for V600E-mutated cases) are recommended [7].

Our case exemplifies the diagnostic complexity of adult HPR-LCH and further demonstrates the efficacy of involved-field IMRT for local control, while also highlighting the critical need for systemic surveillance and subsequent chemotherapy when extracranial progression occurs.

## Case presentation

A 35-year-old female presented with a six-month history of progressive memory decline, characterized by an inability to recall recent events. She was accompanied by polydipsia and polyuria, as well as personality changes marked by irritability. Over the preceding six months, she had gained approximately 15 kg in weight. She also reported amenorrhea for one year, along with intermittent galactorrhea and bilateral nipple erosion. Initial cranial MRI performed at a local hospital revealed symmetrical abnormal signals in the bilateral thalami, mammillary bodies, dorsal brainstem, third ventricle, and periaqueductal gray matter, suggestive of a metabolic disorder. The patient was subsequently admitted to the Department of Neurology at our hospital in February 2025 for further evaluation and management.

Upon admission, laboratory investigations revealed an elevated serum prolactin level of 94.19 ng/mL. Other pituitary hormones and serum cortisol levels were within normal limits. A 24-hour fluid balance assessment demonstrated increased fluid intake (up to 4000 mL/day) and urine output (up to 6000 mL/day). Serum electrolyte analysis revealed hypernatremia (Na⁺ 155.02 mmol/L) and hyperchloremia (Cl⁻ 116.37 mmol/L). Oral desmopressin acetate (0.1 mg three times daily) was initiated, which effectively reduced urine output to below 3000 mL/day and corrected the electrolyte imbalances. Lumbar puncture was performed, and cerebrospinal fluid (CSF) analysis revealed a protein level of 0.78 g/L, chloride level of 138.02 mmol/L, and a normal cell count. Cytological examination of the CSF was negative for malignant cells. Testing for demyelinating antibodies in the CSF was negative. Serum and CSF levels of human chorionic gonadotropin (hCG) and alpha-fetoprotein (AFP) were within normal limits. Chest CT scan showed no significant abnormalities. The patient was treated symptomatically for “metabolic encephalopathy” for eight days, then transferred to our department to further clarify the lesion’s characteristics.

**Fig. 1:**
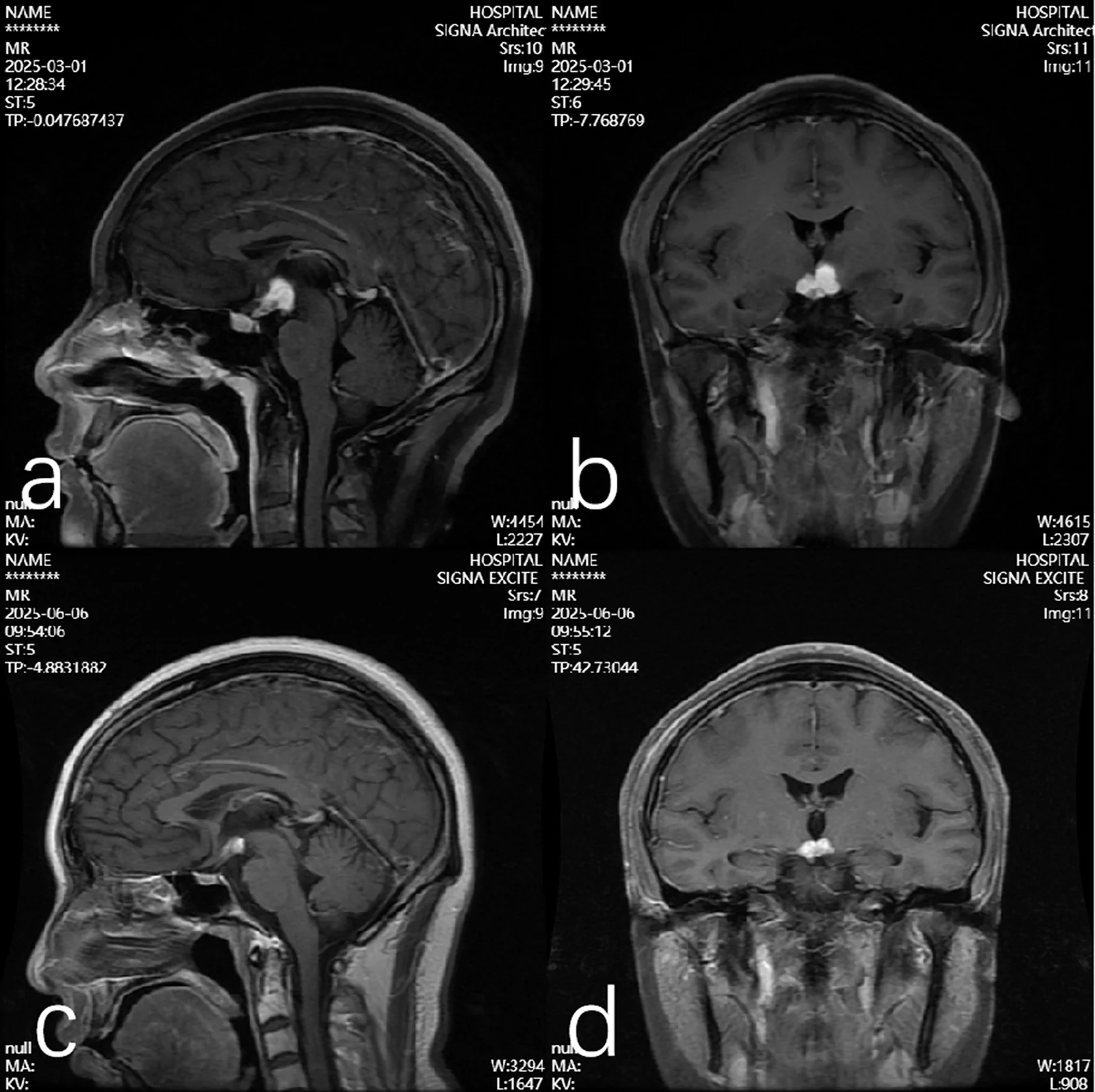
Pre- and post-radiotherapy contrast-enhanced T1-weighted MRI of the hypothalamic-pituitary lesion. **(a**,** b)** Pre-treatment: coronal and sagittal views showing a 2.0 × 1.4 cm enhancing hypothalamic lesion with pituitary stalk thickening and a left-sided pituitary nodule (0.4 × 0.3 cm). **(c**,** d)** Six weeks post-radiotherapy (June 2025): significant reduction in the hypothalamic lesion size with residual enhancement

Following transfer, the patient continued to exhibit significant memory impairment, along with mental sluggishness, reduced speech, and a depressed mood. Cranial MRI revealed a nodular abnormal signal in the hypothalamic region with ill-defined margins, demonstrating iso- to hypointense signal on T1-weighted FLAIR images and high signal intensity with heterogeneity on T2-weighted and T2-weighted FLAIR images. The surrounding area displayed patchy areas of low signal intensity on T1-weighted images and high signal intensity on T2-weighted images, with well-defined borders. The hypothalamic lesion showed nodular enhancement, measuring approximately 2.0 × 1.4 × 1.6 cm. A left-sided pituitary nodule remained hypointense and measured approximately 0.4 × 0.3 cm, with heterogeneous enhancement (Fig. 1a and b).

**Fig. 2:**
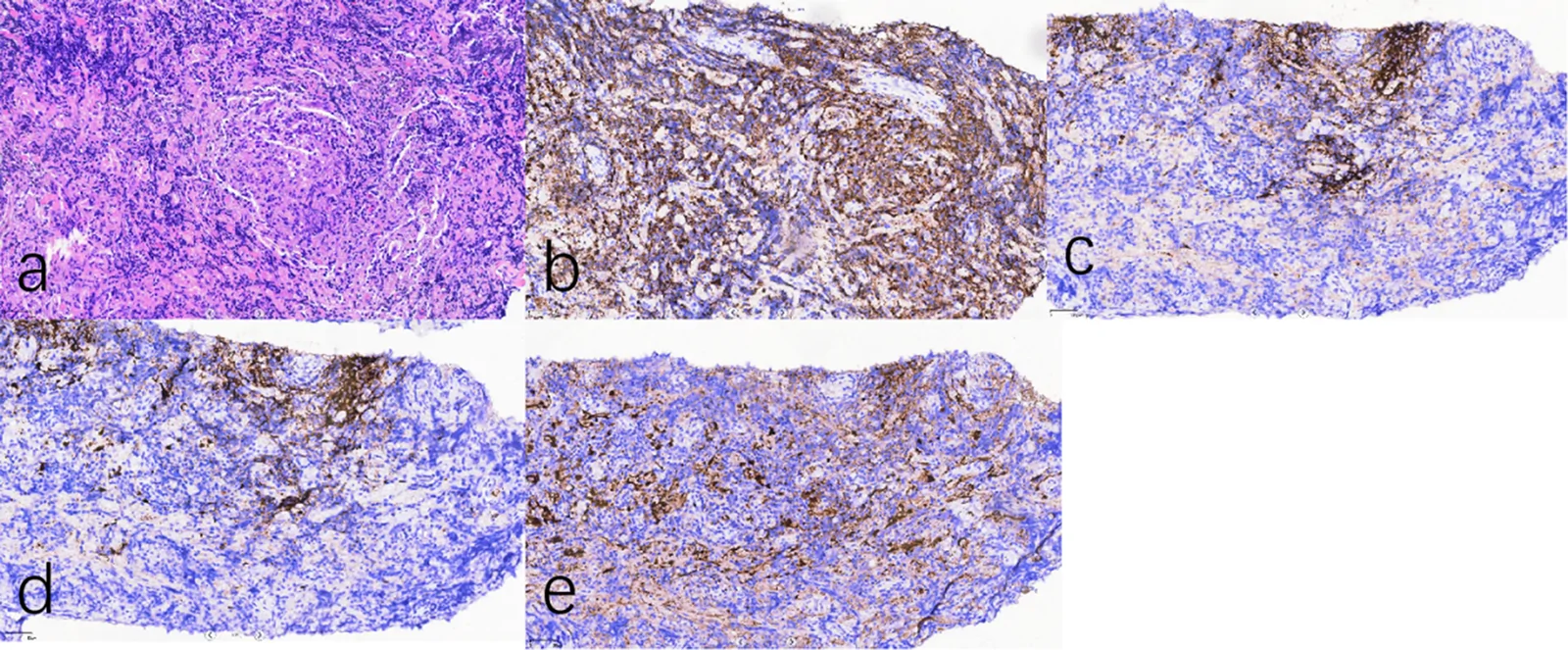
Immunohistochemical confirmation of LCH. (**a**) hematoxylin and eosin staining (HE,×200). (**b**) CD68 positivity (SP×200). (**c**) CD1a positivity (SP×200). (**d**) Langerhans’ cells positivity (SP×200). (**e**) S100 positivity (SP×200)

On March 7, 2025, a right frontotemporal (pterional) craniotomy was performed under general anesthesia for biopsy of the hypothalamic lesion. This approach was chosen because the lesion’s epicenter was located in the hypothalamus and mammillary bodies—supra-sellar structures that are difficult to safely reach via the transsphenoidal route. The pterional approach allowed direct visualization and acquisition of a sufficiently large tissue sample to definitively confirm LCH histology, which is critical for differentiating it from germinoma and lymphocytic hypophysitis. The procedure was uneventful, and the patient recovered without new neurological deficits. Postoperative histopathological examination confirmed the diagnosis of Langerhans cell histiocytosis. Immunohistochemical staining revealed positive results for CD68, S100, CD1a, and Langerin (CD207), with a Ki67 proliferation index of approximately 5% (Fig. 2). Molecular testing for the BRAF V600E mutation using quantitative PCR was negative (Supplementary Fig. 1).

Post-biopsy, the patient’s condition stabilized, and she was discharged in late March 2025. At discharge, before initiating bromocriptine, her prolactin level had decreased to 2.37 ng/mL, which we attributed to relief of pituitary stalk compression following biopsy. However, because the patient continued to experience galactorrhea and nipple erosion, bromocriptine (2.5 mg daily) was started and maintained to control these symptoms. She was instructed to continue oral desmopressin acetate, bromocriptine, and levothyroxine sodium on an outpatient basis. Levothyroxine had been initiated preoperatively based on the diagnosis of central hypothyroidism (low free T4 with inappropriately normal TSH), and was continued because follow-up testing confirmed persistent central hypothyroidism despite radiological improvement.

On April 22, 2025, the patient underwent involved-field intensity-modulated radiotherapy (IMRT) in our Oncology Department with the following parameters: total dose 2200 cGy in 11 fractions, targeting the visible enhancing hypothalamic-pituitary lesion with a 5-mm margin. Organs at risk (optic chiasm, brainstem) were constrained according to QUANTEC guidelines. Follow-up brain MRI performed on June 6, 2025 (6 weeks post-radiotherapy) demonstrated significant reduction in lesion size (Fig. 1c and d), with clinical improvement in polyuria symptoms, evidenced by more stable fluid intake/output balance and reduced thirst, despite continued desmopressin therapy.

### Follow-up and subsequent systemic progression

Following IMRT, the patient’s polyuria was well-controlled with oral desmopressin, and her cognitive symptoms stabilized. However, over the subsequent months, she developed progressive ulcerative skin lesions on the right frontotemporal scalp and persistent bone pain in the right zygomatic and frontal regions. Galactorrhea and bilateral nipple erosion persisted despite bromocriptine therapy. To comprehensively evaluate the disease extent, a whole-body 18 F-FDG PET-CT scan was performed on February 3, 2026 (10 months post-radiotherapy) at the National Cancer Center / Chinese Academy of Medical Sciences. The scan revealed complete metabolic remission of the hypothalamic and third ventricular lesion (no abnormal FDG uptake). However, new multisystem involvement was identified, including: right frontotemporal bone destruction with soft tissue mass (SUVmax 10.0), multiple subcutaneous nodules over the scalp, neck, abdominal wall, and vulva (SUVmax 10.2), bilateral pulmonary nodules (SUVmax 7.6), and cervical/supraclavicular lymphadenopathy (SUVmax 2.9).

In light of this systemic progression, the patient was referred to the Department of Medical Oncology at Peking Union Medical College Hospital. On March 6, 2026, she commenced systemic chemotherapy with high-dose methotrexate (MTX) at an affiliated hospital (Beijing Longfu Hospital). The regimen consisted of a full initial course: MTX 9 g intravenously weekly for 3 weeks, followed by MTX 12 g weekly for 2 weeks, and then MTX 16 g weekly for 2 weeks (totaling 7 weeks of therapy). This initial course was completed in late May 2026 (approximately 3 months after the PET-CT evaluation). Remarkably, after completing the initial MTX course, the patient’s skin ulcers showed near-complete healing, bone pain markedly resolved, and galactorrhea with nipple erosion substantially improved.

At the time of this report (July 2026, 15 months post-radiotherapy and 2 months after completion of the initial MTX course), the patient remains on maintenance desmopressin, levothyroxine, and bromocriptine under regular endocrine surveillance. She is clinically stable, with skin and bone lesions well-controlled, and is preparing for a scheduled second consolidation chemotherapy course in August 2026.

## Discussion and conclusion

Recent studies by Radojkovic et al. [8] and Zhou et al. [9] have highlighted that HPR-LCH often presents with nonspecific neurological symptoms (e.g., memory decline in our case), which frequently leads to delayed diagnosis averaging 6–12 months. Our patient exhibited the classic triad of CDI, hyperprolactinemia, and memory/cognitive dysfunction. This constellation of symptoms, when combined with characteristic MRI findings of pituitary stalk thickening (> 3 mm) and mammillary body enhancement, should raise immediate suspicion for LCH [3, 4]. However, the differential diagnosis for hypothalamic-pituitary lesions is broad and includes germinoma, lymphocytic hypophysitis, IgG4-related disease, and neurosarcoidosis. Germinomas typically present with elevated CSF hCG or AFP and often involve the pineal gland. Lymphocytic hypophysitis more commonly affects women in the peripartum period and shows homogeneous stalk enhancement without mammillary body involvement. IgG4-related disease is often associated with elevated serum IgG4 and other organ involvement (e.g., pancreas, salivary glands). Neurosarcoidosis frequently exhibits leptomeningeal enhancement and pulmonary manifestations. The immunohistochemical confirmation (CD1a+, Langerin+) remains the diagnostic gold standard [6], and in our case, it was essential for establishing the correct diagnosis.

The pathophysiological mechanisms underlying our patient’s symptoms merit discussion. Weight gain (15 kg over six months) likely resulted from ventromedial hypothalamic nucleus involvement, leading to loss of satiety and dysregulated energy homeostasis. Memory decline can be attributed to bilateral mammillary body damage, disrupting the Papez circuit (hippocampus–mammillary bodies–anterior thalamus–cingulate gyrus), which is critical for episodic memory formation.

The data on optimal treatment approaches for adult patients with HPR-LCH are limited. For unifocal disease, surgical excision may be curative if it can be safely undertaken. However, HPR-LCH not amenable to local therapies should be treated similar to multisystem disease with systemic therapies [10]. In systemic therapies, cladribine, higher doses of cytarabine, intravenous methotrexate-based regimens, or kinase inhibitors are preferred [11]. Research conducted by Kriz et al. shows radiotherapy is a very effective and safe treatment option, and even low doses show sufficient local control [12]. Laird et al. also maintained that radiotherapy remains an effective option for treatment of LCH, although lesions in sites such as the brain and skin remain difficult to control with standard low-dose ranges [13].

The dynamic clinical trajectory of our case provides several important therapeutic insights.

First, involved-field IMRT achieved durable local control of the HPR lesion, as confirmed by both conventional MRI and functional PET-CT (showing complete metabolic remission at 15 months). This supports the current consensus that moderate-dose radiotherapy (15–24 Gy) is effective for organ-confined or function-threatening LCH lesions [7, 12, 14]. Notably, our patient’s BRAF V600E-negative status did not preclude an excellent radiotherapeutic response, which is consistent with the hypothesis that radiosensitivity in LCH may be independent of this specific mutation.However, it is important to emphasize that comprehensive molecular profiling was not performed in this patient; therefore, any discussion of alternative MAPK pathway alterations, such as MAP2K1 mutations, remains speculative and should not be interpreted as an established biological explanation for the observed response.

Second, local control does not equate to systemic remission. Despite excellent intracranial response, the patient developed progressive bone, skin, and pulmonary involvement. This underscores the fundamental nature of LCH as a multisystem clonal disorder that requires whole-body surveillance, even when the presenting manifestation appears localized. It also highlights the limitation of relying solely on local therapies for what may be an occult systemic disease.

Third, our case demonstrates the efficacy and tolerability of high-dose methotrexate as a subsequent systemic therapy for BRAF-negative multisystem LCH. While cladribine and BRAF inhibitors are commonly cited options [11], MTX-based regimens have also shown activity in refractory LCH [10]. The marked clinical improvement in skin ulcers and bone pain following the initial MTX course supports its value in this context, particularly when targeted agents are not applicable.

Fourth, the endocrine sequelae—persistent CDI and central hypothyroidism, with galactorrhea only resolving after systemic chemotherapy—highlight the complex interplay between local tumor mass effects, systemic inflammatory mediators, and permanent end-organ damage. Long-term hormone replacement remains the cornerstone of endocrine management, independent of oncological outcomes.

## Limitations

Several limitations should be acknowledged. First, this is a single-case report, and our findings cannot be generalized without validation in larger cohorts. Second, the limited biopsy specimen size precluded comprehensive molecular profiling (e.g., MAP2K1 mutation analysis or immune microenvironment characterization), which could have provided deeper biological insights into the differential response to radiotherapy versus chemotherapy. Third, although we have 15 months of follow-up, the long-term durability of both the radiotherapeutic and chemotherapeutic responses remains to be determined, as late relapses and delayed endocrine deterioration are well recognized in LCH. Fourth, the potential contribution of spontaneous regression or individual biological variability cannot be entirely excluded. Despite these limitations, we believe this case provides valuable real-world evidence for the multidisciplinary management of adult HPR-LCH.

## Conclusion

This case offers three critical clinical lessons for adult HPR-LCH:1.Involved-field IMRT can be a highly effective local treatment for HPR-LCH, achieving durable radiographic and metabolic remission, even in BRAF V600E-negative cases, as demonstrated in this patient.2.Close systemic surveillance is essential. Local response does not predict systemic control; patients must be closely monitored for extracranial progression, and timely systemic chemotherapy (e.g., high-dose MTX) is critical for managing multisystem disease.3.Multidisciplinary management (endocrinology, neurosurgery, radiation oncology, and medical oncology) is strongly recommended to navigate the complex interplay between local tumor control, systemic therapy, and permanent endocrine rehabilitation.

Long-term follow-up and further research are needed to standardize radiotherapy protocols and identify biomarkers predicting progression to multisystem disease in initially HPR-predominant LCH.

## Supplementary Information

Below is the link to the electronic supplementary material.

Supplementary Material 1

## Data Availability

The datasets supporting the conclusions of this article is included within the article and in figures.

## References

[1] Harmon CM, Brown N. Langerhans cell histiocytosis: a clinicopathologic review and molecular pathogenetic update. Arch Pathol Lab Med. 2015;139:1211–4. 10.5858/arpa.2015-0199-RA 26414464

[2] Makras P, Stathi D, Yavropoulou M, Tsoli M, Kaltsas G. The annual incidence of Langerhans cell histiocytosis among adults living in Greece. Pediatr Blood Cancer. 2020;67(9):e28422. 10.1002/pbc.28422 32618036

[3] Brys ADH, Vermeersch S, Forsyth R, Velkeniers B, Bravenboer B. Central diabetes insipidus: beware of Langerhans cell histiocytosis! Neth J Med. 2018;76:445–9. 30569891

[4] Huo Z, Lu T, Liang Z, Ping F, Shen J, Lu J, et al. Clinicopathological features and BRAF(V600E) mutations in patients with isolated hypothalamic-pituitary Langerhans cell histiocytosis. Diagn Pathol. 2016;11:100. 10.1186/s13000-016-0548-5 PMC5070363 27760550

[5] Jinguji S, Nishiyama K, Yoshimura J, Yoneoka Y, Harada A, Sano M, et al. Endoscopic biopsies of lesions associated with a thickened pituitary stalk. Acta Neurochir. 2013;155:119–24. 10.1007/s00701-012-1543-6 23108562

[6] Oda Y, Amano K, Seki Y, Kimura S, Yamashita K, Masui K, et al. Clinical features and difficulty in diagnosis of Langerhans cell histiocytosis in the hypothalamic-pituitary region. Endocr J. 2022;69(4):441–9. 10.1507/endocrj.EJ21-0341 34776469

[7] Goyal G, Tazi A, Go RS, Rech KL, Picarsic JL, Vassallo R, et al. International expert consensus recommendations for the diagnosis and treatment of Langerhans cell histiocytosis in adults. Blood. 2022;139(17):2601–21. 10.1182/blood.2021014343 PMC11022927 35271698

[8] Radojkovic D, Pesic M, Dimic D, Radjenovic Petkovic T, Radenkovic S, Velojic-Golubovic M, et al. Localised Langerhans cell histiocytosis of the hypothalamic-pituitary region: case report and literature review. Hormones. 2018;17:119–25. 10.1007/s42000-018-0024-6 29858859

[9] Zhou W, Rao J, Li C. Isolated Langerhans cell histiocytosis in the hypothalamic-pituitary region: a case report. BMC Endocr Disord. 2019;19:143. 10.1186/s12902-019-0474-0 PMC6924050 31856773

[10] Haupt R, Minkov M, Astigarraga I, et al. Langerhans cell histiocytosis (LCH): guidelines for diagnosis, clinical work-up, and treatment for patients till the age of 18 years. Pediatr Blood Cancer. 2013;60(2):175–84. 10.1002/pbc.24367 PMC4557042 23109216

[11] Agaimy A, Bonert M, Naqvi A, et al. Langerhans cell histiocytosis associated with renal cell carcinoma is a neoplastic process: clinicopathologic and molecular study of 7 cases. Am J Surg Pathol. 2020;44(12):1658–65. 10.1097/PAS.0000000000001562 32910018

[12] Kriz J, Eich HT, Bruns F, Heyd R, Schäfer U, Haverkamp U, et al. Radiotherapy in Langerhans cell histiocytosis – a rare indication in a rare disease. Radiat Oncol. 2013;8:233. 10.1186/1748-717X-8-233 PMC3852286 24103738

[13] Laird J, Ma J, Chau K, Chelius M, Shi W, Zhang Z, et al. Outcome after radiotherapy for Langerhans cell histiocytosis is dependent on site of involvement. Int J Radiat Oncol Biol Phys. 2018;100(3):670–8. 10.1016/j.ijrobp.2017.10.053 PMC5806138 29413280

[14] Tan H, Yu K, Yu Y, An Z, Li J. Isolated hypothalamic-pituitary Langerhans’ cell histiocytosis in female adult. Medicine. 2019;98(2):e13853. 10.1097/MD.0000000000013853 PMC6336579 30633157

