# Supplementary material for: From local control to systemic management: radiotherapy and sequential methotrexate therapy in an adult BRAF-negative hypothalamic-pituitary Langerhans cell histiocytosis – a case report with 15-month follow-up
Source: BMC Endocr Disord. 2026 Aug 25;26:376. doi: 10.1186/s12902-026-02509-2 (PMC13769157; doi:10.1186/s12902-026-02509-2)
Supplement: Supplementary file 1 [file 12902_2026_2509_MOESM1_ESM.docx]

**Supplementary Fig. 1** Quantitative PCR amplification plot for BRAF V600E mutation detection. No amplification signal was observed for the mutant allele, confirming BRAF V600E-negative status. The wild-type control and positive control are shown for comparison.
